# Healthcare workers’ perspectives on barriers and facilitators to referral and recruitment to diabetes prevention programmes: a systematic review protocol

**DOI:** 10.12688/hrbopenres.13702.2

**Published:** 2024-03-28

**Authors:** Clair Haseldine, Gráinne O'Donoghue, Patricia M Kearney, Fiona Riordan, Sarah Cotterill, Sheena McHugh

**Affiliations:** 1 School of Public Health, University College Cork, Cork, Ireland; 2 School of Public Health, Physiotherapy and Sports Science, University College Dublin, Dublin, Ireland; 3 Health Service Executive, Cork, Ireland; 4 School of Health Sciences, University of Manchester, Manchester, UK

**Keywords:** Systematic review, diabetes prevention programme, implementation, healthcare workers’ perspective, “best fit” framework synthesis, Theoretical Domains Framework

## Abstract

**Background:**

Diabetes is a growing global health problem. International guidelines recommend identification, screening, and referral to behavioural programmes for those at high risk of developing type 2 diabetes. Diabetes prevention programmes (DPPs) can prevent type 2 diabetes in those at high risk, however many eligible participants are not referred to these programmes. Healthcare workers (HCWs) are pivotal to the referral and recruitment processes. This study aims to identify, appraise and synthesise the evidence on barriers and facilitators to referral and recruitment to DPPs from the perspective of HCWs.

**Methods:**

A “best fit” framework synthesis method will synthesise qualitative, quantitative, and mixed methods evidence on factors that affect HCWs referral and recruitment to DPPs, with the Theoretical Domains Framework (TDF) as the *a priori* framework. MEDLINE, EMBASE, CINAHL, PsychINFO, Web of Science and Scopus will be searched for primary studies published in English. Year of publication will be restricted to the last 26 years (1997–2023). Quality will be assessed using the Mixed Methods Appraisal Tool. A mix of deductive coding using the TDF and inductive coding of data that does not fit the TDF will be synthesised into themes representing the whole dataset. The relationships between the final set of themes will be explored to create a new model to understand HCWs’ perspectives on referral and recruitment to DPPs. Sensitivity analysis will be carried out on this conceptual model. Confidence in the synthesised findings will be assessed using the GRADE-CERQual approach. One author will screen, extract, appraise the literature while a second author will independently verify a 20% sample at each stage.

**Discussion:**

Participation in DPPs is key for programme impact. HCWs typically identify those at risk and refer them to DPPs. Understanding HCWs’ perspectives on the barriers and facilitators to referral and recruitment will inform future implementation of DPPs.

## Introduction

Diabetes is a growing global health problem that places considerable burden on individuals and health systems ^1^. Type 2 diabetes accounts for over 90% of all cases of diabetes with 438 million prevalent cases worldwide in 2019 ^2, 3^. The prevalence and the death rates from type 2 diabetes rose by 49% and 11% respectively from 1990 to 2019 ^2^. This rising prevalence of type 2 diabetes has been attributed to ageing populations, improving medical care (which leads to people living longer with diabetes) and the rising incidence of diabetes risk factors, especially obesity ^4^.

The Corona virus disease 2019 (Covid 19) pandemic was particularly devastating for people with diabetes. The risk of severe disease or death from Covid 19 in those with type 2 diabetes was at least twice as high than for those without diabetes ^5^. Diabetes increased the burden of Covid 19 on health systems and economies globally ^4^ with evidence that the risk of developing type 2 diabetes is increased after Covid 19 infection even in those who were at low risk or had a mild infection ^6^. The predicted rise in type 2 diabetes prevalence combined with the potential further increase in the incidence of type 2 diabetes due to Covid 19 infection has given rise to calls for urgent action to tackle the problem ^4^.

Similar to type 2 diabetes, the prevalence of prediabetes is also rising globally ^7^. Prediabetes is a stage of raised blood glucose levels where people at high risk of developing diabetes can be identified through measurement of fasting blood glucose, glycated haemoglobin or 2-hour post prandial load ^7^. The International Diabetes Federation estimated 7.5% of people (374 million) worldwide live with impaired glucose tolerance ^8^ and identifying people at high risk is essential in tackling the growing problem of type 2 diabetes. Landmark randomised controlled trials investigating the effect of lifestyle change on diabetes incidence in those at high risk have demonstrated that type 2 diabetes can be prevented ^9– 12^. A meta-analysis by Galaviz *et al.* in 2018 synthesising the global impact of 63 diabetes prevention programmes (DPPs) that were implemented under real-world conditions or translated from proven interventions found that people receiving a lifestyle intervention had a 29% lower risk of developing type 2 diabetes than people who did not receive one ^1^. DPPs aim to prevent type 2 diabetes by changing dietary and physical activity behaviours by raising awareness of the change needed, setting dietary and physical activity goals, action planning and relapse prevention ^13^.

International guidelines recommend identification, screening, and referral to DPPs for those at high risk of developing type 2 diabetes ^13, 14^. However, many eligible participants are not referred to or do not participate in DPPs when recruited ^15, 16^. A study investigating reach and use of diabetes prevention services in the USA found that only 4.9% of people at high risk were referred to a DPP and less than half (39.6%) of those attended ^17^. The national DPP in England reports uptake by the first 100,000 referred to the programme was 56%, with only 19% completing it ^18^. In a scoping review of existing evidence of the National Health Service DPP in England, the lack of evidence on how to improve initial engagement with the programme was highlighted and efforts to improve recruitment of those of working age, those from deprived and ethnic minority backgrounds was recommended ^19^.

The reasons for low rates of referral and recruitment to DPPs are complex and multifaceted. System level factors can include a lack of engagement by leadership and a poor fit of the programme within the existing organisation ^20^. Healthcare workers play a critical role in the referral pathway ^21^. In a systematic review examining the implementation of DPPs, Aziz *et al.* (2015) suggests that high risk people identified and referred by health professionals resulted in higher participation rates, highlighting the importance of the healthcare worker in the referral process ^22^. Studies have highlighted how healthcare professionals influence a person’s view of their diagnosis of prediabetes and their decision to participate in DPP programmes ^17, 23^. Furthermore, a 2022 meta-synthesis on the barriers and facilitators to lifestyle change from the perspective of those at risk of type 2 diabetes reported that the guidance and education given by healthcare professionals facilitated positive change ^24^. In a 2017 systematic review that explored the factors affecting diabetes prevention in the primary care setting, healthcare professionals were concerned about the extra workload that identifying people at risk of type 2 diabetes would bring and the impact on resources ^25^. Some identified their lack of knowledge about preventing diabetes and a lack of confidence in providing diabetes prevention advice as barriers to providing preventative services. While most referrals to DPPs come through health professionals, not all DPPs are delivered in primary care. The national DPP in England for example is delivered by providers outside the national health service ^26^. In the United States DPPs are delivered by community-based organisations, pharmacies, as well as health clinics ^27^ and programmes are available online ^28^. The perspective of this broader group on referral and recruitment to DPPs warrants further investigation.

Therefore, this review aims to identify, appraise and synthesise the evidence on barriers and facilitators to referral and recruitment to DPPs from the perspective of healthcare workers (HCWs). Referral and recruitment to DPPs are the target behaviours. This review will adopt a “best fit” framework synthesis, a pragmatic and flexible approach, which integrates theory into systematic reviews ^29^. The framework was primarily applied in the analysis stage and it did not inform data extraction. The “best fit” framework synthesis uses an existing theoretical framework (in this case the Theoretical Domains Framework) to organise the extracted data thereby ensuring the researcher is engaging with theory throughout the review process ^30^. The “best fit” framework has the flexibility to consider themes outside of the *a priori* framework which allows for the theoretical framework to be tested and refined if necessary to better encapsulate the evidence available ^29^.

## Methods

This protocol describes a systematic review of qualitative, quantitative and mixed methods research on HCWs’ perspectives on factors that affect referral and recruitment to DPPs. The systematic review has been prospectively registered on 21/12/2022 with the International Prospective Register of Systematic Reviews (PROSPERO) (CRD42022383023). This protocol adheres to the Preferred Reporting Items for Systematic review and Meta-Analysis Protocols (PRISMA-P) reporting guidelines ^31, 32^.

### Review design

A “best fit” framework approach will be used to synthesise the evidence ^29^ and the Preferred Reporting Items for Systematic review and Meta-Analysis (PRISMA) guidelines ^33^ will be followed for reporting. The “best fit” framework synthesis method is flexible, pragmatic and is suitable for describing or explaining decisions about health and health behaviours ^29^. The selection of this approach was informed by the RETREAT framework (Review question, Epistemology, Time, Resources, Expertise, Audience and purpose, Type of data) ^34^.

This review follows the seven steps of the “best fit” framework ^29^ ( Table 1).

**Table 1.** Worked example of the “best fit” framework ^29^.

Step 1: Identifying a research question	Research Question: What are HCWs perspectives on the barriers and facilitators to referral and recruitment to DPPs?	
Step 2a: Identify framework Step 2b: Information sources and search strategy	(a) RETREAT ^34^ framework informed the choice of the TDF ^36^ as the a priori framework	(b) Systematically identify research studies using SPIDER framework ^35^
Step 3: Screening, data extraction, quality assessment	Extract data and appraise quality using the Mixed Methods Appraisal Tool version 2018 ^37^	
Step 4: Deductive coding	Code the extracted data to the TDF	
Step 5: Inductive coding	Develop new themes for the data that does not fit the domains of the TDF	
Step 6: Develop the “best fit” framework	Develop a new framework from the TDF and the new themes	
Step 7: Assess the potential for bias in the new model/framework	Explore the relationships between the TDF and the new themes to create a new model to understand HCWs’ perceptions of barriers and facilitators to referral and recruitment in DPPs.	

HCWs: Healthcare Workers, DPPs: Diabetes Prevention Programmes, TDF: Theoretical Domains Framework

**Step 1:** Identifying a research question

An initial scoping search was conducted to inform the research question and aims. The question was refined using the SPIDER (Sample, Phenomenon of Interest, Design, Evaluation and Research) framework ^35^. This framework was adapted from the PICO (Population, Intervention, Comparison, Outcome) tool to facilitate the inclusion of qualitative and mixed methods as well as quantitative studies in reviews ^35^.

**Research question**: What are HCWs’ perspectives on the barriers and facilitators to referral and recruitment to DPPs? 

**Eligibility criteria:** The eligibility criteria for this study are informed by the SPIDER framework ( Table 2) ^35^.

**Table 2.** Eligibility criteria.

SPIDER	Inclusion	Exclusion
S: Sample	HCWs’ involved in referral or recruitment to DPPs	Perspectives of participants or people at high risk of diabetes
P of I: Phenomenon of Interest	Studies that report referral or recruitment to DPPs from HCWs’ perspectives	Studies that include HCWs’ perspectives on referral to programmes for type 2 diabetes or gestational diabetes
D: Design	Qualitative, quantitative and mixed methods research designs	
E: Evaluation	Experiences, views, perspectives, beliefs, opinions, factors (barriers and enablers) that affect referral or recruitment to DPPs	
R: Research	Qualitative, quantitative and mixed methods in English published from January 1997 to March 2023	Editorials, opinion pieces or commentaries

HCWs: Healthcare Workers, DPPs: Diabetes Prevention Programmes

**Sample:** Any HCW involved in identifying, screening, referring or recruiting people at high risk of diabetes to DPPs. This could include, but not limited to, professionals such as primary care physicians, practice nurses, pharmacists, physiotherapists, dietitians, public health nurses, hospital doctors and nurses and could also include educators on the DPPs such as community health workers and lifestyle coaches. In line with the WHO definition, which encompasses support personnel, HCWs could also include administrative staff or management who may have knowledge of the referral or recruitment process ^38^. Studies that include the participants’ perspectives on DPPs will only be included if data from the perspective of the HCW can be extracted separately.

**Phenomenon of Interest:** Studies that report HCWs’ perspectives on referral or recruitment to DPPs will be included. The perspective could include barriers and facilitators to recruitment and referral. A barrier will be defined as any factor that obstructs or impedes referral or recruitment to DPPs. A facilitator will be defined as any factor that promotes or eases referral or recruitment to DPPs. The barriers and facilitators can also include HCWs’ perspectives of broader social and environmental influences. The DPPs should be programmes aimed at decreasing body weight if appropriate, achieving dietary recommendations and achieving physical activity recommendations. The programmes should be aimed at adults at high risk of developing type 2 diabetes. As this is a study on HCWs’ opinions and not on the effectiveness of the programme the definition of high risk will not be limited to those with non-diabetic hyperglycaemia but could include those identified as being at high risk on a diabetes risk score ( *e.g.,* FINDRISK) or being overweight or obese ^39^. There will be no restriction based on the setting, intensity or delivery mode of the programme. Studies of HCWs’ perspectives on programmes for type 2 diabetes or gestational diabetes will be excluded.

**Design and Research Type:** All primary qualitative, quantitative and mixed methods research that meet the eligibility criteria will be included ( Table 2). These could include qualitative ( *e.g.,* case studies or grounded theory), quantitative ( *e.g.,* cross sectional studies) or mixed methods studies which combine qualitative and quantitative data collection and analysis. Data collection methods could include observations, interviews, focus groups, surveys and questionnaires. The year of publication will be restricted to studies published after 1997 as this was when the first landmark trial on diabetes prevention was published ^9^. Only studies published in English will be included due to the limited time available to conduct the review and limited access to translation services.

**Step 2a:** Identify framework

The “best fit” approach allows for either identifying an appropriate framework from the published literature to guide the evidence synthesis or for generating a new meta-framework by systematically searching for and synthesising published frameworks. This study will use the former approach, selecting the Theoretical Domains Framework (TDF) as the *a priori* framework ^36, 40^. This framework was chosen as it is a synthesis of systematically reviewed behaviour change theories, developed specifically to understand and inform the implementation of evidence-based interventions ^36, 40^. The TDF is a comprehensive and rich source of theory that has been used previously in primary studies to understand implementation in DPPs ^41^ and it has been used as the *a priori* framework in the “best fit” method in a systematic review of patients’ perceptions of diabetes prevention and cardiovascular disease programmes ^42^.

**Step 2b:** Information sources and search strategy

Systematic searching of electronic databases will be combined with supplementary search methods ( *i.e.,* reference and citation searching) to increase the identification of relevant research ^43^. The databases commonly used in diabetes prevention systematic reviews will be searched. These include MEDLINE, EMBASE, CINAHL, PsychINFO, Web of Science and Scopus. A university librarian was consulted to further refine the search strategy. The search will use database specific controlled vocabulary ( *e.g.,* medical subject headings- MeSH), as well as key words in title and abstract, spelling variants, truncation and synonyms. Search dates (January 1997–March 2023). An example of a MEDLINE search is provided in Table 3.

**Table 3.** Example of a MEDLINE search strategy.

Search No.	Search Terms
1	Exp Diabetes Mellitus, type 2
2	Prediabetic state
3	S1 OR S2
4	(Prediabet* OR pre-diabet* OR hypergly* OR “Impaired fasting glucose” OR “Impaired glucose tolerance” OR IGT OR IFG OR “prediabetic state” OR (diabet* N4 risk)) ti,ab
5	S3 OR S4
6	Exp Preventative health services
7	Exp Health education
8	Exp Life style
9	S6 OR S7 OR S8
10	(diabet* N4 prevent*) N2 (program* OR service* OR intervention*) ti,ab OR (lifestyle OR “life style”) N2 (change* OR program* OR intervention* OR modif*) ti,ab
11	S9 OR S10
12	S5 AND S11
13	Exp Health personnel
14	Community health workers
15	S16 OR S17
16	("health* professional*" OR doctor* OR physician* OR nurse* OR "general practitioner" or GP* OR clinician* OR "health* worker" OR pharmacist* OR physiotherapist* OR "physical therapist*" OR dietitian* OR nutrition* OR "lifestyle coach*" OR "community health worker*" OR "health coach*" OR educator* OR “practice manager” OR “practice staff” OR practitioner*) OR ((family OR health OR healthcare OR “health care” OR medical OR nursing OR nutrition OR pharmacy OR physiotherapy OR “physical therapy” OR “public health” OR trainee) N2 (assistant* OR director* OR manager* OR officer* OR personnel OR practice OR practitioner* OR professional* OR provider OR staff)) ti,ab
17	S15 OR S16
18	S12 AND S17
19	Exp Attitude
20	attitude* OR perception* OR opinion* OR view* OR belief* OR barrier* OR facilitator* OR enabler*
21	S19 OR S20
22	S18 AND S21

**Step 3:** Screening

All references will be imported to Covidence, a systematic review software to manage screening, data extraction and quality assessment. If this software were unavailable an open access free alternative such as Rayyan could be used. Duplicates will be removed. At the beginning of the screening process the lead author and a second author will screen a small number of studies and meet to iteratively discuss their screening decisions, reflect on any differences of opinion and resolve any disagreements. A third author will be consulted if consensus cannot be reached. The lead author will then screen the abstract and titles of the remaining studies and the second reviewer will screen a random sample of 20% of the articles. Studies that do not meet the eligibility criteria will be excluded. If there is any doubt about exclusion at the title and abstract stage the studies will be kept in until the full text stage. The full text of all the potentially eligible research papers will then be read by the lead author to see if they meet the criteria to be included in the review. A second author will review a random sample of 20% of the articles. The lead author and a second reviewer will meet to iteratively discuss decisions and a third author will be consulted if required to reach consensus. A PRISMA flow diagram will be used to illustrate the screening and inclusion process and will outline the reasons for exclusion at the full text stages ^33^.

**Step 3 (continued):** Data extraction

Verbatim quotes and authors’ interpretations relating to HCWs’ perceptions of barriers and facilitators to recruitment and referral will be extracted from the results and discussion sections using a data extraction form. The data extracted will note whether it is a direct quote or author interpretation. Analysis from quantitative and mixed methods studies, such as survey results and descriptive summaries will also be extracted. Two authors will pilot this form with three of the included studies (one qualitative, one quantitative and one mixed methods if available) and the form will be refined and modified as needed with input from the wider research team if necessary ^29^. The lead author will then extract the data from all included studies while the second author will extract the data from a random sample of 20% of the studies. Both authors will meet to iteratively discuss their decisions and a third author will be consulted if agreement cannot be reached.

The data extraction form will contain the following sections: study information ( *e.g.,* study number, title, first author and year of publication), characteristics of the study ( *e.g.,* aims, research design, sampling and sample size), participant characteristics ( *e.g.,* age, gender, years qualified, profession), programme characteristics ( *e.g.,* frequency, mode of delivery, length of programme, programme participants, setting (e.g. clinic, hospital, community), socioeconomic context) data collection approach and method of analysis and reported barriers and facilitators or related themes. If multiple reports from the same study are found, then these will be linked to avoid extracting the same data more than once.

**Step 3 (continued):** Quality assessment

The quality of the studies will be appraised using the Mixed Methods Appraisal Tool ^44^. This tool contains five different criteria which allows for concomitant appraisal of qualitative research, randomised controlled trials, non-randomised quantitative studies, descriptive quantitative studies and mixed methods studies. The tool has been piloted for reliability and efficiency ^45, 46^ and was updated in 2018 ^37^. The 2018 version recommends three steps to screen, choose the appropriate category and to rate the criteria. Studies will not be excluded if rated as low quality as they may have important insights ^47^. The lead author will perform the quality assessment on all included studies while a second author will assess a random 20% sample. This will be an iterative process whereby three studies (qualitative, quantitative and mixed methods if available) will be assessed by each author and notes on decisions will be compared. This iterative process will continue with further studies until both authors are mostly in agreement to ensure a similar approach to the task. A third author will be consulted if necessary to resolve any disputes.

**Step 4 and step 5:** Deductive and inductive coding 

The data extracted from the studies on the barriers and facilitators to referral and recruitment will be coded deductively against the TDF Version 2 ^36^. This version has 14 theoretical domains and 84 component constructs related to behaviour change. The lead author will develop a coding tree in Nvivo with guidance from the 14 TDF domains and the 84 constructs. If this software were unavailable, analysis could be conducted using Excel and Word or open access software such as Taguette. Data that do not fit into the 14 TDF domains will be coded to an ‘other’ code and secondary thematic analysis of these codes will be performed ^29^. An example of determinants coded to the “other” domain could be HCWs’ perspectives of broader social and environmental factors which limit their patient’s ability to participate. To ensure consistency in analysis three studies (qualitative, quantitative and mixed methods if available) will be jointly coded by the lead author and a second author. Quantitative data on the perceptions of HCWs will be coded to the relevant TDF domains. All data will be thematically synthesised. Quantitative data may be maintained in the reporting of results. Both authors will discuss their coding decisions and any discrepancies that cannot be resolved by consensus will be discussed with a third author. The lead author will conduct the analysis on the remaining studies and a second author on 20% of the articles.

**Step 6:** Develop the “best fit” framework

The new themes will be added to the TDF domains, and an adapted model will then be produced by synthesising the *a priori* themes from the TDF and the additional concepts identified ^29^.

**Step 7:** Assess the potential for bias in the new model/framework

Relationships between the themes will be explored to create a new model or framework to understand the barriers and facilitators to recruitment and referral to DPPs from the HCWs’ perspective. Differences between the TDF and the new framework ( *i.e.,* the absence or addition of themes) will be explored to assess and explain the selection and reporting of the research ^29^. This will highlight if the differences are justifiable or if the literature needs to be reviewed again. If there is no evidence of dissonance in the synthesis, then purposeful efforts will be made to seek out “negative” cases ^48^. Any sensitivity of the new framework to the study design, setting, quality assessment, subgroups of HCWs, year of data collection, delivery mode, and location will be examined. This will be conducted by examining the effect of excluding studies ( *e.g.,* those assessed as being low quality) on the themes in the framework.

### Confidence

Confidence in the synthesised findings will be assessed using the GRADE-CERQual (Grades of Recommendation, Assessment, Development and Evaluation-Confidence in Evidence from Reviews of Qualitative research) approach ^49^. The outcome of interest will be confidence in the evidence for barriers and facilitators to referral and recruitment to DPPs from the HCWs’ perspective. The assessment of confidence will be based on four components: any limitations in the methodology of the primary studies contributing to a finding, how relevant the review question is to the review finding, how coherent a review finding is, and how adequate the data supporting a review finding is ^49^. Ratings of confidence (high, moderate, low or very low) will be assigned to each review finding. Two authors will be involved in the confidence assessment and the review team will be consulted if further input is required to reach consensus.

### Outcomes

The outcomes will be barriers or facilitators to referral or recruitment to DPPs from the HCWs’ perspective.

### Ethics

Ethical approval is not required for systematic reviews

### Study status

The study has not yet commenced. The planned start for this study is March 2023.

## Conclusion/Discussion

The potential of diabetes prevention programmes to impact on type 2 diabetes has not been fully realised due to the low numbers of people referred to and participating in the programmes. HCWs are a vital part of the referral pathway and can influence how seriously a person views their risk and their decision to take part. Using theory to understand the HCWs perspectives can give insights into why referral to DPPs is not routine practice and can identify what needs to be changed to improve referral and recruitment. This review will develop a conceptual framework to provide a broader understanding of these behaviours and will move beyond the description of the data to try to understand the relationships between the barriers and enablers identified. Using the “best fit” framework with the TDF as the *a priori* framework ensures that important themes are not overlooked while also allowing for new themes to be developed. The “best fit” framework is flexible and pragmatic allowing for the integration of qualitative, quantitative and mixed methods data. The inclusion of different forms of evidence will strengthen this review as “complex questions demand complex forms of evidence” ^50^.

The findings of this review will be relevant to health systems and policy makers who have developed or are developing diabetes prevention programmes and will support further implementation efforts to improve recruitment and referral to DPPs.

## Data Availability

No data are associated with this article. Figshare: PRISMA-P checklist for “Healthcare workers’ perspectives on barriers and facilitators to referral and recruitment to diabetes prevention programmes: a systematic review protocol”. https://doi.org/10.6084/m9.figshare.22141151 ^32^. Data are available under the terms of the Creative Commons Attribution 4.0 International license (CC-BY 4.0).
