# Supplementary material for: Healthcare workers’ perspectives on barriers and facilitators to referral and recruitment to diabetes prevention programmes: a systematic review protocol
Source: HRB Open Res. 2024 Mar 28;6:23. Originally published 2023 Apr 6. [Version 2] doi: 10.12688/hrbopenres.13702.2 (PMC11004599; doi:10.12688/hrbopenres.13702.2)
Supplement: Peer review history

# Reviewer report: Reviewer response for version 2

**DOI:** 10.21956/hrbopenres.15226.r38896

**Published:** 2024-04-09

**Authors:** Radhika Gore

**Affiliations:** 1 NYU School of Medicine, New York, USA

I have no further comments.

Is the study design appropriate for the research question?

Partly

Is the rationale for, and objectives of, the study clearly described?

Yes

Are sufficient details of the methods provided to allow replication by others?

Yes

Are the datasets clearly presented in a useable and accessible format?

Not applicable

Reviewer Expertise:

I have expertise in implementation science research, qualitative methods, and the application of social science theory to health services and public health research questions. I have published on health care workers’ perspectives on chronic disease management and prevention, including diabetes prevention, in diverse primary care settings.

I confirm that I have read this submission and believe that I have an appropriate level of expertise to confirm that it is of an acceptable scientific standard.

# Reviewer report: Reviewer response for version 1

**DOI:** 10.21956/hrbopenres.14987.r37353

**Published:** 2024-01-30

**Authors:** Louise Foley

**Affiliations:** 1 University of Limerick, Limerick, County Limerick, Ireland

This protocol paper presents a comprehensive and well-justified plan for conducting a systematic review and framework synthesis of the evidence on barriers and facilitators to referral and recruitment to diabetes prevention programmes from the perspective of healthcare workers. The protocol is clearly presented and addresses an important step in the process of diabetes prevention, with anticipated implications for future implementation of such prevention programmes. Minor suggestions are offered below for the authors’ consideration.

**Introduction:**

- While it is implied that DPPs involve behaviour change, the content of these programmes could be briefly described in the introduction section to provide more context on what individuals are being asked to do when referred/recruited.
- It is stated that the “best fit” method integrates theory into all parts of the systematic review and it seems theory is most explicitly integrated at the analysis/synthesis stage. Did theory also inform the development of the research question and identification of relevant studies, and if so was this based on guidance for applying the TDF?

**Methods:**

- The TDF will provide a comprehensive framework for coding data on barriers and facilitators to DPP referral. As a behaviour change framework has been selected to structure the analysis, further attention could be given to the behavioural perspective in the justification for its use. For example, is referral and recruitment to DPPs positioned as the target behaviour, and if so is the overarching aim of this review to identify potential determinants of the current and desired behaviour? Being more explicit about the behavioural aspect will strengthen the justification for using the TDF here and may also help to guide the coding process.
- In keeping with the “best fit” method, the protocol is framed with the assumption that new themes will be generated to account for data that cannot be deductively coded within the domains of the TDF. While the secondary thematic analysis will be conducted inductively, can the authors provide some suggestions as to how the TDF might be limited for coding relevant barriers and facilitators, for example are determinants beyond behavioural determinants anticipated to be relevant to recruitment and referral to DPP?
- Challenges with coding data to TDF domains have been reported previously, including challenges defining the boundaries of domains and instances where data points appear to fit within several domains ^1,2^. Will the coding tree be developed at the level of the 14 domains, or will it also refer to the 84 component constructs?
- The approach to coding mixed and quantitative data to the TDF domains could be described in further detail. Will quantitative data be maintained in the reporting of results and if so, how will this be synthesised with qualitative evidence in the TDF coding and/or secondary thematic analysis?

Is the study design appropriate for the research question?

Yes

Is the rationale for, and objectives of, the study clearly described?

Yes

Are sufficient details of the methods provided to allow replication by others?

Yes

Are the datasets clearly presented in a useable and accessible format?

Not applicable

Reviewer Expertise:

health psychology, self-management, health behaviour change

I confirm that I have read this submission and believe that I have an appropriate level of expertise to confirm that it is of an acceptable scientific standard.

## References

[1] : A guide to using the Theoretical Domains Framework of behaviour change to investigate implementation problems. *Implement Sci* .2017;12(1) : 10.1186/s13012-017-0605-9 77 10.1186/s13012-017-0605-9 28637486 PMC5480145

[2] : Understanding effects in reviews of implementation interventions using the Theoretical Domains Framework. *Implement Sci* .2015;10: 10.1186/s13012-015-0280-7 90 10.1186/s13012-015-0280-7 26082136 PMC4469259

# Response

**Published:** 2024-03-25

**Authors:** clair haseldine

**Affiliations:** UCC, Ireland

Dear Reviewer,

 Thank you very much for your very helpful review. We made every effort to address all the points raised. 

**Reviewer 2**

**Introduction**

 1.While it is implied that DPPs involve behaviour change, the content of these programmes could be briefly described in the introduction section to provide more context on what individuals are being asked to do when referred/recruited.

**Response**

 1.Thank you for this suggestion. A brief description of the content of DPP has been added to the introduction section.

**Reviewer 2**

**Methods**

 The TDF will provide a comprehensive framework for coding data on barriers and facilitators to DPP referral. As a behaviour change framework has been selected to structure the analysis, further attention could be given to the behavioural perspective in the justification for its use.

 3. For example, is referral and recruitment to DPPs positioned as the target behaviour, and if so is the overarching aim of this review to identify potential determinants of the current and desired behaviour?  Being more explicit about the behavioural aspect will strengthen the justification for using the TDF here and may also help to guide the coding process.

**Response**

 3. Referral and recruitment to DPPs are the target behaviours. This review aims to identify HCWs perspectives on the barriers and enablers to referral and recruitment. This has now been clarified in the introduction.

**Reviewer 2**

 In keeping with the “best fit” method, the protocol is framed with the assumption that new themes will be generated to account for data that cannot be deductively coded within the domains of the TDF.

 4. While the secondary thematic analysis will be conducted inductively, can the authors provide some suggestions as to how the TDF might be limited for coding relevant barriers and facilitators, for example are determinants beyond behavioural determinants anticipated to be relevant to recruitment and referral to DPP?

**Response**

 4. While the TDF will provide a framework to understand the influences on the HCWs behaviour it may not capture the HCWs perceptions on the broader social and environmental issues as highlighted by reviewer 1. An example of this could be the HCWs perception of financial constraints which limit their patient’s ability to participate in DPPs. An example has been added to **Step 4 and 5.**

**Reviewer 2**

 5. Challenges with coding data to TDF domains have been reported previously, including challenges defining the boundaries of domains and instances where data points appear to fit within several domains1,2. Will the coding tree be developed at the level of the 14 domains, or will it also refer to the 84 component constructs?

 5. The coding tree will refer to the 14 domains and the 84 constructs for guidance. This is now clarified in **Step 4 and 5**.

**Reviewer 2**

 6. The approach to coding mixed and quantitative data to the TDF domains could be described in further detail. Will quantitative data be maintained in the reporting of results and if so, how will this be synthesised with qualitative evidence in the TDF coding and/or secondary thematic analysis?

 6. Quantitative data on the perceptions of HCWs will be coded to the relevant TDF domains. All data will be thematically synthesised. Quantitative data and may be maintained in the reporting of results.

 This has been clarified in **Step 4 and 5.**

**Abstract**

 The abstract contained 1 error for the year of publication which has been corrected and now reads “Year of publication will be restricted to the last 26 years (1997–2023)”.  2 typos in the abstract were corrected.

# Reviewer report: Reviewer response for version 1

**DOI:** 10.21956/hrbopenres.14987.r35019

**Published:** 2023-08-24

**Authors:** Radhika Gore

**Affiliations:** 1 NYU School of Medicine, New York, USA

Thank you for the opportunity to review this article. The protocol is well-motivated with evidence on the rising prevalence of type 2 diabetes, role of lifestyle modification in preventing diabetes, low referral rates to diabetes prevention programs (DPPs), and role of health care workers (HCWs) in facilitating referrals. Understanding the barriers and facilitators to DPP referral and recruitment from HCWs’ perspectives is an important goal, and the research question is clearly stated. My comments mainly focus on whether and how the review process and findings will illuminate social and environmental aspects of barriers and facilitators to DPP referral and recruitment. I discuss and encourage the authors to consider some of the pathways of influence that may be implicated here, and I offer suggestions relevant to their phenomenon of interest and theoretical framework.

 The authors rightly note that reasons for low rates of referral and recruitment are complex. I draw out particular insights from the evidence they cite to motivate attention to social and environmental influences. For instance, the literature points to the role of structural factors (e.g., “outer setting” from the Consolidated Framework for Implementation Research or CFIR) in influencing DPP implementation, and it highlights that such factors are not widely surfaced in the literature (Madrigal *et al.*, 2022 ^1^). Evidence shows how HCWs perceive and respond, or feel unable to respond, to the social determinants of patients’ health in relation to prediabetes management (Barry and Greenhalgh, 2022 ^2^). Relatedly, a 2017 systematic review (Messina *et al.*, 2017 ^3^), although focused on primary care settings, presents three themes broadly relevant for the delivery and uptake of diabetes prevention interventions. The themes cover the role of context and setting, including features of the healthcare setting and perceived responsibility for lifestyle change; the role of patient factors such as motivation, including social determinants such as financial or time constraints that place diabetes prevention resources out of patients’ reach; and the role of professional factors, e.g., workload and time (including competing health priorities), self-efficacy, knowledge, and perceptions of patients’ motivation.

 Taken together, this background literature suggests complex direct and indirect effects of social and environmental factors on HCWs’ perceptions and behavior. A potential illustrative pathway involving social and environmental factors could be the following: Structural, external “outer setting” factors and patients’ social determinants of health can influence patient motivation, which can inform HCWs’ perceptions about patients’ behavior and likely receptiveness to DPPs, which in turn can influence (and reinforce) HCWs’ decisions and actions in counseling and referrals for diabetes prevention. “Outer setting” factors can also directly influence HCWs’ behavior and perspectives on DPP referrals and recruitment.

 While the Theoretical Domains Framework (TDF) is an appropriate choice for an *a priori* framework, it would likely provide a close understanding of reflective, psychological processes that inform HCWs’ perspectives and behavior (Atkins *et al.*, 2017 ^4^). The TDF does include “social influences” and “environmental context and resources” as constructs (Atkins *et al.*, 2017 ^4^), yet as defined in the TDF these refer to domains of influence on HCWs’ behavior. My concern is that analysis based on the TDF alone may not adequately capture and distinguish social and environmental influences on patient behavior, which in turn can affect HCWs’ perceptions and behavior. Can the authors discuss whether and how their review will address this? To what extent and how will the TDF be used to analyze social and environmental influences on HCWs’ behavior and perspectives?

 Examining social and environmental influences is significant not only because the social determinants of health are associated with risk of diabetes (Hill-Briggs *et al.*, 2020 ^5^). Social factors are additionally salient here because the review will examine the perspectives of a wide range of HCWs. HCWs’ awareness of patients’ social circumstances and sense of responsibility to address patients’ social needs and lifestyle change can vary by their role, professional identity, and service setting, which can shape HCWs’ perspectives and actions with regard to DPP referrals. An outcome of low DPP referrals may be differently explained by different mechanisms depending on the type of HCW. For example, community health workers may understand their role as encompassing support and assistance to resolve patients’ social needs (Islam *et al.*, 2017 ^6^ and Malcarney *et al.*, 2017 ^7^). They may expend effort on activities related to social needs and could delay or defer DPP referrals and counseling on health behavior. Physicians in resource-constrained, safety-net health care settings may have little time for health counseling within clinical encounters and may feel compelled to prioritize patients with diabetes rather than those at risk for diabetes (Gore *et al.*, 2020 ^8^). They may fail to direct patients at risk for diabetes to (limited) resources such as community health workers who could refer patients to diabetes prevention programs. Can the authors discuss how the TDF will be applied to analyze the views and experiences of diverse subgroups of HCWs within the target population?

 The “best fit” approach would lend flexibility and capture themes that lie outside the TDF. However, an extension or a complementary theoretical framework (Atkins *et al.*, 2017 ^4^) – one option is the Consolidated Framework for Implementation Research (CFIR) -- could be useful from the outset to guide analysis of and elaborate social-environmental factors that shape HCWs’ perspectives.

 Another reason to account for social and environmental factors is to be alert to gaps in the data. A relevant and notable finding from the 2017 systematic review examining the delivery and uptake of diabetes prevention interventions was that socio-demographic data – such as on patients’ income, ethnicity, and education -- were poorly reported in its reviewed literature (Messina *et al.*, 2017 ^3^). This made it difficult to fully understand the context behind study findings (Messina *et al.*, 2017 ^3^).

 A final point. In describing their phenomenon of interest, the authors state that they will seek studies that report, from HCWs’ perspective, “the experience of a barrier or facilitator to referral or recruitment to DPPs.” This framing of the phenomenon of interest, namely the HCWs’ experience of a barrier or facilitator, seems to imply that HCWs attempt to make referrals and are unsuccessful or successful based on barriers and facilitators. Yet evidence suggests that low referral rates are partly due to factors such as HCWs’ workload, lack of awareness, and competing health priorities in providing care. These factors indicate in part HCWs’ inability to initiate referrals in the first place; they may not be attempting to make DPP referrals at all. While the research question is clear -- “What are HCWs perspectives on the barriers and facilitators to referral and recruitment to DPPs?” -- the phenomenon of interest appears to comprise several elements: HCWs’ behavior with respect to DPP referrals; HCWs’ experiences of barriers and facilitators when making referrals; HCWs’ perspectives on barriers and facilitators, whether or not they make or attempt to make DPP referrals.

 A reference to HCWs’ behavior is present in the Conclusion section as well. The research question is focused on HCWs’ *perspectives *on barriers and facilitators to DPP referrals and recruitment, but in the Conclusion/Discussion section the authors refer to HCWs’ *behavior*: “Using theory to understand the HCWs behaviour can give insights into why referral to DPPs is not routine practice …” The proposed review is likely to provide insights into both behavior and perspectives, i.e., what HCWs do in terms of DPP referrals and HCWs’ explanations for and views on why they do or don’t make DPP referrals. Can the authors clarify the scope and content of the phenomenon of interest with regard to the question of perspectives and behavior?

Is the study design appropriate for the research question?

Partly

Is the rationale for, and objectives of, the study clearly described?

Yes

Are sufficient details of the methods provided to allow replication by others?

Yes

Are the datasets clearly presented in a useable and accessible format?

Not applicable

Reviewer Expertise:

I have expertise in implementation science research, qualitative methods, and the application of social science theory to health services and public health research questions. I have published on health care workers’ perspectives on chronic disease management and prevention, including diabetes prevention, in diverse primary care settings.

I confirm that I have read this submission and believe that I have an appropriate level of expertise to confirm that it is of an acceptable scientific standard, however I have significant reservations, as outlined above.

## References

[1] : Inner and outer setting factors that influence the implementation of the National Diabetes Prevention Program (National DPP) using the Consolidated Framework for Implementation Research (CFIR): a qualitative study. *Implement Sci Commun* .2022;3(1) : 10.1186/s43058-022-00350-x 104 10.1186/s43058-022-00350-x 36183133 PMC9526531

[2] : How do UK general practice staff understand and manage prediabetes? A focus group study. *BJGP Open* .2022;6(2) : 10.3399/BJGPO.2021.0166 10.3399/BJGPO.2021.0166 35523431 PMC9447313

[3] : A narrative systematic review of factors affecting diabetes prevention in primary care settings. *PLoS One* .2017;12(5) : 10.1371/journal.pone.0177699 e0177699 10.1371/journal.pone.0177699 28531197 PMC5439678

[4] : A guide to using the Theoretical Domains Framework of behaviour change to investigate implementation problems. *Implement Sci* .2017;12(1) : 10.1186/s13012-017-0605-9 77 10.1186/s13012-017-0605-9 28637486 PMC5480145

[5] : Social Determinants of Health and Diabetes: A Scientific Review. *Diabetes Care* .2020;44(1) : 10.2337/dci20-0053 258-79 10.2337/dci20-0053 33139407 PMC7783927

[6] : Evaluating community health workers' attributes, roles, and pathways of action in immigrant communities. *Prev Med* .2017;103: 10.1016/j.ypmed.2017.07.020 1-7 10.1016/j.ypmed.2017.07.020 28735724 PMC6354577

[7] : The Changing Roles of Community Health Workers. *Health Serv Res* .2017;52 Suppl 1(Suppl 1) : 10.1111/1475-6773.12657 360-382 10.1111/1475-6773.12657 28127766 PMC5269544

[8] : Integrating Community Health Workers into Safety-Net Primary Care for Diabetes Prevention: Qualitative Analysis of Clinicians' Perspectives. *J Gen Intern Med* .2020;35(4) : 10.1007/s11606-019-05581-8 1199-1210 10.1007/s11606-019-05581-8 31848857 PMC7174477

# Response

**Published:** 2024-03-25

**Authors:** clair haseldine

**Affiliations:** UCC, Ireland

Dear Reviewer,

 Thank you so much for your very helpful review. We made every effort to address all the points raised.

**Reviewer 1**

 My concern is that analysis based on the TDF alone may not adequately capture and distinguish social and environmental influences on patient behavior, which in turn can affect HCWs’ perceptions and behavior.

 1.Can the authors discuss whether and how their review will address this?

 2. To what extent and how will the TDF be used to analyze social and environmental influences on HCWs’ behavior and perspectives?

**Response**

 1.Thank you for your very helpful insights and suggestions. The “best fit” framework can address the concerns that you raise about the TDF by allowing for consideration of the broader social and environmental issues that may not be adequately reflected in the TDF. These factors can be included in the “other” domain. This perspective has been added to the Phenomenon of Interest section in the methods.

 2. To facilitate understanding of the social context the data extraction form ( **Step 3**: data extraction) will now collect information on the programme participants, and the setting (e.g. clinic, hospital, community) and the socioeconomic context. Relationships between factors will be examined to understand how these broader factors can affect HCWs perceptions and referrals.

**Reviewer 1**

 3. Can the authors discuss how the TDF will be applied to analyze the views and experiences of diverse subgroups of HCWs within the target population?

**Response**

 3. Where specific HCWs groups can be isolated the dominant themes from these studies will be considered. This has been clarified in **Step 7**.

**Reviewer 1**

 4. The “best fit” approach would lend flexibility and capture themes that lie outside the TDF. However, an

 extension or a complementary theoretical framework (Atkins *et al.*, 2017 ^4^ ) – one option is the Consolidated Framework for Implementation Research (CFIR) -- could be useful from the outset to guide analysis of and elaborate social-environmental factors that shape HCWs’ perspectives.

**Response**

 4. Thank you very much for this suggestion. A recent study combined the TDF and CFIR and found that the combination can assist with the interpretation of findings in implementation research (O’Donovan et al., 2023). However, the authors also note that using both frameworks had implications for project management and timelines. A 2017 systematic review examining the combined use of the TDF and CFIR found using both frameworks could introduce unnecessary complexity. (Birkin et al., 2017). In light of the changes made to capture the HCWs views of the social and environmental factors affecting their patients (response 1 above) we are optimistic that the “best fit” approach will be flexible enough to capture the themes outside of the TDF and acknowledge that the project timeline will not accommodate the inclusion of another framework at this stage. The strengths and limitations of the fit of the TDF and the factors coded to the “other” domain will be considered in the discussion.

**References:**

 O’Donovan, B., Kirke, C., Pate, M., McHugh, S., Bennett, K., & Cahir, C. (2023). Mapping the Theoretical Domain Framework to the Consolidated Framework for Implementation Research: do multiple frameworks add value?. *Implementation Science Communications*, 4(1), 100.

 Birken, S. A., Powell, B. J., Presseau, J., Kirk, M. A., Lorencatto, F., Gould, N. J., ... & Damschroder, L. J. (2017). Combined use of the Consolidated Framework for Implementation Research (CFIR) and the Theoretical Domains Framework (TDF): a systematic review. *Implementation science,* 12, 1-14.

**Reviewer 1**

 5. Another reason to account for social and environmental factors is to be alert to gaps in the data. A relevant and notable finding from the 2017 systematic review examining the delivery and uptake of diabetes prevention interventions was that socio-demographic data – such as on patients’ income, ethnicity, and education -- were poorly reported in its reviewed literature (Messina et al., 2017). This made it difficult to fully understand the context behind study findings (Messina et al., 2017).

**Response**

 5. To identify any gaps the data extraction form will now collect information on the programme participants, the setting (e.g. clinic, hospital, community) and the socioeconomic context.

**Reviewer 1**

 6. Can the authors clarify the scope and content of the phenomenon of interest with regard to the question of perspectives and behavior?

**Response**

 6. Thank you for highlighting this. The phenomenon of interest has now been clarified and the word “experience” removed to allow for perspectives other than those that HCWs have directly experienced such as workload, lack of awareness and competing health priorities. In the conclusion the word “behaviour” has been removed and “perspective” has been added to clarify that the phenomenon of interest is the HCWs perspective.
